# Magnesium Intake and Sleep Disorder Symptoms: Findings from the Jiangsu Nutrition Study of Chinese Adults at Five-Year Follow-Up

**DOI:** 10.3390/nu10101354

**Published:** 2018-09-21

**Authors:** Yingting Cao, Shiqi Zhen, Anne W. Taylor, Sarah Appleton, Evan Atlantis, Zumin Shi

**Affiliations:** 1 School of Medicine, University of Adelaide, SAHMRI, L7, North Terrace, Adelaide, SA 5000, Australia; anne.taylor@adelaide.edu.au (A.W.T.); E.Atlantis@westernsydney.edu.au (E.A.); zumin.shi@adelaide.edu.au (Z.S.); 2 Jiangsu Provincial Centre for Disease Control and Prevention, Nanjing 210000, China; cdczsq@163.com; 3 The Health Observatory, University of Adelaide, Queen Elizabeth Hospital Campus, Woodville, SA 5000, Australia; sarah.appleton@adelaide.edu.au; 4 School of Nursing and Midwifery, Western Sydney University, Sydney, NSW 2000, Australia; 5 Human Nutrition Department, Qatar University, Doha 00000, Qatar

**Keywords:** dietary magnesium, daytime falling asleep, Chinese adults

## Abstract

(1) Background: In clinical trials, dietary magnesium use can improve insomnia symptoms. However, little is known about the association between dietary magnesium consumption and sleep disorder symptoms including daytime falling asleep, sleepiness and snoring at the population level. (2) Methods: We used data from 1487 adults aged 20 and above attending the Jiangsu Nutrition Study. At baseline in 2002, dietary magnesium was assessed by 3-day weighed food records. At follow-up in 2007, sleep disorder symptoms, including daytime falling asleep, sleepiness and snoring at night, were gathered using a sleep questionnaire. (3) Results: The mean intake of magnesium was 332.5 mg/day. In total, 5.3%, 13.2% and 35.7% of the subjects reported daytime falling asleep, daytime sleepiness, and snoring during sleep, respectively. Compared with the lowest quartile of magnesium intake, the highest quartile was associated with decreased likelihood of falling asleep (odds ratio (OR) 0.12 (0.02, 0.57)) in women but not in men after adjusting for demographic, anthropometric, lifestyle factors, hypertension, and overall dietary patterns. No associations were found between dietary magnesium intake and daytime sleepiness nor night snoring in either gender. (4) Conclusions: Dietary magnesium intake may have long-term benefits in reducing the likelihood of daytime falling asleep in women.

## 1. Introduction

Sleep and wake cycle is regulated by the suprachiasmatic nucleus in the brain, which also coordinates the circadian rhythms in other tissues through the body [1]. Poor quantity and quality of sleep have been suggested to be adversely associated with a range of metabolic and cardiovascular systems, as well as quality of life [2,3]. Impaired sleep quantity and quality of sleep may be manifested as sleep disorder symptoms including daytime sleepiness, daytime falling asleep, and snoring at night. An increasing number of studies has found the associations between dietary macronutrients consumption and sleep outcomes. For example, carbohydrate with high glycemic index facilitated sleep onset, while low fiber, high saturated fat and sugar intake are associated with lighter and less restorative sleep with more arousals [4,5,6]. Sleep duration is positively associated with consumption of iron and zinc, but negatively associated with vitamin K and B12 [7]. However, the research of micronutrients and sleep is limited, and the findings are inconsistent.

Magnesium, a cofactor involved in more than 300 enzyme systems, regulates diverse biochemical reactions in the body [8]. Recently, magnesium has also been found to regulate cellular timekeeping in both animal and plant cells [9], thus it is beneficial to maintain the normal circadian rhythms and ensure a quality sleep in humans. Magnesium supplement use improves insomnia symptom among older people in a double-blind placebo-controlled clinical trial [10]. Low dietary magnesium intake was found to be significantly associated with depression [11]. It is known that depression is associated with poor sleep. However, the studies on dietary magnesium and sleep are limited.

The major dietary sources of magnesium include green leafy vegetables, whole grains, nuts, and legumes. These foods products are relatively inexpensive in China where average daily intakes are high compared to Western countries. We hypothesized that high intake of magnesium is beneficial for the prevention of sleep disorder symptoms including daytime falling asleep, sleepiness and snoring. Therefore, the present study aimed to investigate the associations between baseline dietary magnesium intake and sleep symptoms at follow-up using five-year longitudinal data from the Jiangsu Nutrition Study.

## 2. Materials and Methods

### 2.1. Subjects

**Figure 1:**
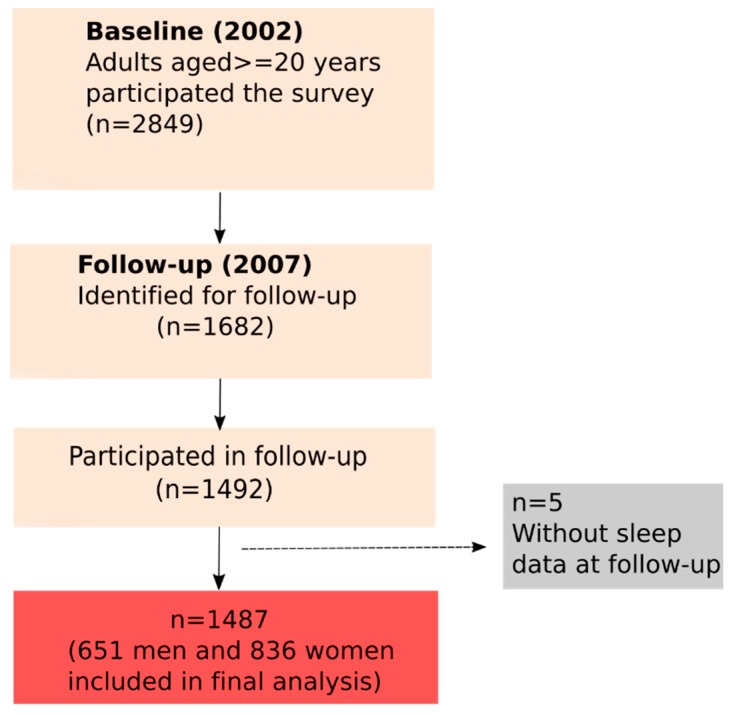
Sample description from Jiangsu Nutrition Study.

Data from the Jiangsu Nutrition Study cohort were used in the current study. Detailed methodology has been described previously [12]. In brief, in 2002, 2849 adults at least 20 years old living in two cities and six rural areas in Jiangsu Province took part in the Chinese National Nutrition and Health Survey. In 2007, an attempt to recontact all 1628 original participants was made and 1492 participated undertook the follow-up interview. For the current analysis, we included only those subjects with baseline (in 2002) magnesium intake and sleep records at follow-up (in 2007) (*n* = 1487) (Figure 1). The study was conducted according to the guidelines in the Declaration of Helsinki, and all procedures were approved by the Jiangsu Provincial Centre for Disease Control and Prevention. Informed consent for participation was obtained from each participant.

### 2.2. Data Collection and Measurements

Participants were interviewed at their homes by trained health workers using a pre-coded questionnaire. Interviews took about 2 h to complete and included questions on diet, sociodemographic information, medical history, cigarette smoking, physical activity, and other lifestyle factors.

### 2.3. Dietary Measurements

Dietary data including magnesium intake were obtained by food weighing plus consecutive individual 3-day food records (including one weekend) that recorded all foods consumed by everyone in the household [13]. At the beginning and end of the 3-day survey, investigators weighed all the food stocked in the household. All purchases, home production, and processed snack foods were weighed and recorded each day. Nutrient intake was calculated using the Chinese Food Composition Table [14].

### 2.4. Sleep Measurement

At baseline in 2002, only sleep duration was collected but both sleep duration and self-reported daytime falling asleep were collected at follow-up in 2007. Self-reported sleep disorder symptoms were determined by relevant questions. Daytime falling asleep was determined by the question “Do you fall asleep involuntarily during the day? (1) no; (2) yes; (3) don’t know”; Daytime sleepiness was assessed by the question “Do you feel sleepy during the day? (1) never; (2) occasionally; (3) sometimes; (4) often and (5) frequently”; and snoring during the night was determined by the question ”Does your partner complain that you snore during the night? (1) never; (2) occasionally; (3) sometimes; (4) often; (5) frequently”.

### 2.5. Anthropometric Measurement and Other Variables

In both 2002 and 2007 anthropometry was conducted using standard protocols and techniques. Body weight was measured in light indoor clothing without shoes to the nearest 0.1 kg. Height was measured without shoes to the nearest mm using a stadiometer. Body mass index (BMI) was calculated as weight in kilogram divided by the square of height in meters. BMI was subsequently categorized into four groups underweight (<18.5 kg/m^2^), normal weight (18.5–23.9 kg/m^2^), overweight (24–27.9 kg/m^2^) and obese (≥28 kg/m^2^) respectively according to the guideline for Chinese adults [15]. Waist circumference was measured to the nearest mm midway between the inferior margin of the last rib and the crest of the ilium, in the mid-auxiliary line in a horizontal plane. Blood pressure was measured twice with a mercury sphygmomanometer on the right upper arm of the subject, who was seated for 5 min before the measurement. The mean of these two measurements was used in the analyses. Hypertension was defined as a systolic blood pressure above 140 mmHg and/or a diastolic blood pressure above 90 mmHg or the use of antihypertensive drugs.

### 2.6. Covariates

The following covariates were included in the study: cigarette smoking (yes or no); alcohol consumption (never, 1–2/week, 3–4/week and daily); education (low, medium and high); occupation (manual or non-manual); sedentary activity (<1 h/day, 1–2 h/day; 2–3 h/day, and ≥3 h/day); residence (urban and rural).

### 2.7. Statistical Analysis

Descriptive data are presented as the mean (SD) (95% (confidence interval (CI)) or as percentage. Chi-square tests were used to compare the differences between categorical variables, and (Analysis of variance) ANOVA was performed to compare differences in continuous variables between groups. Magnesium intake (mg) was recoded into sex-specific quartiles. A sex-specific association between baseline quartiles of magnesium intake and self-reported sleep disorder symptoms at follow-up was assessed using multivariable logistic/Poisson regression models. A set of models were used: model 1 adjusted for age, energy intake; model 2 further adjusted for income, education, region, smoking, alcohol consumption, and sedentary activity; model 3 further adjusted for BMI, hypertension, and diabetes; model 4 further adjusted for four dietary patterns. These four dietary patterns are: Factor 1 (‘macho’) included various kinds of animal foods and alcohol; Factor 2 (the ‘traditional’ pattern) loaded heavily on rice, fresh vegetables and inversely on wheat flour; Factor 3 (‘sweet tooth’) contained cake, milk, yoghurt and drinks; and, Factor 4 (‘vegetable rich’ pattern) was characterized whole grains, fruits, root vegetables, fresh and pickled vegetables, milk, eggs and fish. The four factors explained 28.5% of the variance in intake (published earlier [16]). In sensitivity analyses, we further adjusted for anemia, and baseline sleep duration. Statistical significance was considered when *p* < 0.05. All statistical analyses were performed with STATA 14 (Stata Corporation, College Station, TX, USA).

## 3. Results

In total, 1487 participants had complete dietary intake data and sleep data in both 2002 and 2007. There was a decrease in sleep duration (mean = 0.36 h/day) on average over five years in the study population with an increased sleep duration among short sleepers and a decreased sleep duration among long sleepers. The prevalence of self-reported sleep disorder symptoms at follow-up (year 2007) were: daytime sleepiness 13.2%, falling asleep 5.3% and snoring 35.7%. The median intake of dietary magnesium was 289 mg/day (interquartile range (IQR) 233.7–366.1 mg/day) at baseline (year 2002, mean intake 332.5 mg/day). Sex-specific characteristics by quartiles of magnesium intake at baseline are presented in Table 1. Dietary magnesium intake seemed to decrease as income increased in both sexes. Subjects in manual occupation and from rural region were more likely to consume high level of dietary magnesium. However, non-alcohol consumers were more likely to be low magnesium consumers in men, while those who had more frequent alcohol consumption were more likely to have more dietary magnesium. While in women, compared with the lowest quartile (Q1) of magnesium intake, those who had the highest quartile (Q4) were more likely to have lower education level. With the increase of magnesium intake, the intake of carbohydrate, protein and fat increased significantly in both genders. 

For sleep disorder symptoms at follow-up, there was no difference across quartiles of magnesium intake at baseline except sleep duration and falling asleep in women. Those in Q1 of magnesium intake were more likely to have short sleep duration (<7 h/day) and more likely to fall asleep during the day compared with those who had Q4 of magnesium intake (Table 2). Across quartiles of magnesium intake, the prevalence of falling sleep was 4.9%, 8.6%, 5.0% and 3.7% in men (*p* = 0.240); 8.7%, 4.4%, 5.3% and 1.9% in women (*p* = 0.018), respectively. The prevalence of snoring was similar across quartiles of magnesium in men and women, respectively. After adjusting for age and energy intake, magnesium intake was inversely associated with falling asleep in women but not in men. Across quartiles of magnesium intake, the odds ratio (OR) for falling asleep were 1.00, 0.34 (95% CI 0.14–0.87), 0.42 (95% CI 0.16–1.09) and 0.11 (95% CI 0.02–0.57), respectively in women; 1.00, 1.32 (0.69, 2.50), 1.39 (0.68, 2.83), 1.39 (0.68, 2.83), and 1.62 (0.69, 3.80) respectively in men in the final model (Table 3). There were no associations between quartiles of magnesium consumption and other sleep related symptoms including snoring and daytime sleepiness in either men or women. Sensitivity analyses for further adjustment for anemia and sleep duration at baseline did not change the results (data not shown). Compared to model 1, the subjects lost in the final model (model 4) had no differences in age, and BMI, but with a shorter sleep duration at baseline (*p* < 0.05, data not shown).

## 4. Discussion

Dietary magnesium consumption was found to be associated with decreased risk of falling asleep in women but not in men in the present study. No associations were found between dietary magnesium consumption and other sleep related symptoms in either sex.

Dietary magnesium consumption in the Chinese population has been studied previously. In the Shanghai Women’s Health Study, the median consumption of dietary magnesium was 267 mg/day, which is similar with our result (289 mg/day) in both sexes [17]. However, both values are below the recommended daily amount by China Nutrition Society (330 mg/day) [18]. In the study of comparing nutrients consumption between Chinese and Italians, Japanese and Americans, mean dietary magnesium consumption in Chinese subjects was higher than the Japanese in the younger group and lower than the Americans adults and Italian children and adolescents [19]. The authors claimed that the Chinese diet has been shifting away from the traditional diet towards a diet that contains high-fat, low-carbohydrate, fiber and mineral nutrients.

It is interesting that the magnesium consumption was inversely associated with income and positively associated with manual occupation and rural regions. This is consistent with the high dietary magnesium consumption in rural areas found in a middle-aged Chinese population (mean daily consumption 371.5 mg/day vs 332.5 mg/day in our study with mixed population) [20]. The possible explanation may be the easy access to green leafy vegetables in rural areas and relatively low cost of such foods compared to urban areas.

Our finding of an inverse association between magnesium consumption and daytime falling asleep in women was consistent with other population studies. For example, magnesium consumption has been suggested to improve insomnia in a double-blind placebo-controlled clinical trial in elderly [10]. Improved sleep included subjective sleep efficiency, sleep time, sleep onset latency, and early morning awakening, and increased serum melatonin and decreased serum cortisol concentration in experimental group (500 mg supplemental magnesium daily) compared with the placebo group. Data from National Health and Nutrition Examination Survey (NHANES)suggested a U-shape association between dietary magnesium consumption and sleep duration [21]. This may indicate a potential benefit of increased dietary magnesium consumption on poor quality sleep including both short and long sleep. We also found that the number of short sleepers seemed to decrease across the quartile of dietary magnesium consumption at follow-up, particularly in women. A shortened sleep at night may lead to falling asleep during the day. However, when we further adjusted for short sleep, the association between dietary magnesium consumption and falling asleep in women remained, and even seemed to be stronger. This may suggest that the association is independent of short sleep.

The inverse association between magnesium consumption and falling asleep is biologically plausible. In animal studies, researchers have demonstrated that magnesium deficiency could affect normal sleep-wake rhythm by increasing periods of wakefulness and reducing slow wave sleep in rats, and such effect was recovered when reintroducing magnesium in their diet [22]. Magnesium deficiency has been suggested to related to muscle cramps, which can contribute to a poor sleep and consequent sleepiness the day after. Having magnesium may reduce night time wakefulness and maintain a normal sleep structure, which may explain a beneficial effect of preventing falling asleep during the day found in our study. In the study mentioned above that found a sleep improving effect of magnesium supplements in the elderly, the researchers also showed that magnesium supplementation increased the serum levels of melatonin and renin while reducing cortisol levels [10]. This may partly explain the beneficial effect of magnesium consumption on sleep quality that is via increasing the secretion of melatonin and lowering the level of cortisol. In addition, magnesium consumption has been suggested to be inversely associated with depression in human studies including cross-sectional [11] and longitudinal [23] designs. In animals, removal of magnesium from the diet of mice appears to result in anxiety and depressive-related behavior [24]. An aid of anti-depressant effect of magnesium also guarantees a quality sleep, which reduces poor sleep related sleep disorder symptoms.

The association between magnesium consumption and falling asleep was limited to women. This gender difference is intriguing. Increasing evidence has supported the solid sex differences in terms of biological and physiological differences between men and women (e.g., distinct hormonal and physical changes in women’s life span including puberty, pregnancy, and menopause), which may underlie the differences in sleep physiology and sleep disorders between sexes [25]. In addition, sex differences in terms of nutrition and supplements have been suggested, in which women do not immediately response to a normal increase in dietary carbohydrate when expressed as a percentage of total energy consumption compared with men [26]. Indeed, it has been suggested that dietary consumptions are different in men and women [27], and a Mediterranean dietary intervention had more pronounced benefits in the long term in men than in women [28]. This evidence provides foundations for future gender specific dietary interventions.

Depression has been discussed above as a potential mechanism of understanding the association between magnesium consumption and sleep disorder symptoms, but it remains to be explored whether gender difference in depression can explain the different effects of magnesium consumption on sleep remains to be explored. Earlier studies have reported that women had twice the lifetime risk of developing depression compared with men [29]. Another earlier finding demonstrated that women exhibited a higher prevalence of somatic depression (e.g., body aches, fatigue, and sleep disturbance) than men but not a higher prevalence of pure depression [30]. In a Chinese cross-sectional study that investigated the prevalence and correlates with sleep disturbances and depression in adolescents [31], a higher likelihood of sleep disturbance was found in girls, which was associated with depressive symptoms. These studies indicate a female are inclination for depression, which is particularly associated with somatic symptoms including sleep disturbances.

Magnesium supplement use has been shown to be effectively reduce depressive symptoms in a randomized clinical trial [32]. As magnesium may have a potential anti-depressant effect, magnesium consumption in this particular depressed (including sleep problems) female group may be more beneficial compared with men. Such difference may potentially explain the gender difference in the relationship of magnesium consumption with falling asleep found in our study. However, we do not have depression data, and further exploration of whether depression is a confounder or a mediator in terms of the association between magnesium consumption and daytime falling asleep cannot be conducted.

In addition, it is noticed that in the study sample, we have previously shown that the magnesium consumption is inversely associated with anemia. The prevalence of anemia in women in our study population is almost double that of men [33], and magnesium consumption was also found to be inversely associated the risk of anemia in our study population. Anemia has been shown to be associated with poor sleep quality [34], and the possible mechanism was partly due to the dopaminergic function of iron, which plays an important role in sleep regulation. Whether the gender difference found in our study is related with anemia which women are inclined to have is unknown. Further adjustment for anemia did not change the association between magnesium consumption and sleep disorder symptoms (data not shown).

The strengths of this study include the large general population sample and consecutive three-day weighed food diaries that provide robust food consumption data. Several limitations though need to be acknowledged: (1) supplemental magnesium was not included, mainly because the difficulties of accurate assessment due to variety of brands, and different concentration etc. The number of participants using supplements is very small at baseline; (2) detailed dietary consumption using the same 3-day food record method at follow-up is not available; (3) sleep disorder symptoms were self-reported, which may have bias; however, objective measures such as overnight sleep studies are not feasible to implement in large sample sizes; (4) self-reported sleep disorder symptoms were not collected at baseline. Consequently, we cannot make a conclusion on a causal relationship due to study design.

## 5. Conclusions

In conclusion, dietary magnesium consumption was inversely associated with falling asleep during the day in women but not in men. No associations were found between dietary magnesium consumption and daytime sleepiness or snoring at night. A sex difference in sleep management may need to be considered in future research and practice.

## Figures and Tables

**Table 1:** Sex-specific characteristics of subjects by quartiles of magnesium consumption at baseline (2002) (*n* = 1487) ^1^.

	Quartiles of Magnesium Consumption (mg/day)									
	Men					Women				
Factors	Q1 (*n* = 163)	Q2 (*n* = 163)	Q3 (*n* = 163)	Q4 (*n* = 162)	*p*-Value	Q1 (*n* = 209)	Q2 (*n* = 209)	Q3 (*n* = 209)	Q4 (*n* = 209)	*p*-Value
Nutrients consumption										
* Magnesium consumption (mg/day)	219 (30.3)	288 (16.4)	350 (20.6)	505 (122)	<0.001	186 (27.7)	244 (13.1)	301 (21.4)	462 (132)	<0.001
	(124–259)	(259–315)	(315–392)	(392–1015)		(79–222)	(222–267)	(267–339)	(339–1157)	
Carbohydrate (g/day)	259 (62.1)	317 (63.0)	353 (86.3)	427 (101)	<0.001	219 (56.5)	277 (60.7)	318 (65.9)	392 (102)	<0.001
Protein (g/d)	61.3 (13.9)	73.5 (14.9)	84.3 (15.6)	101.0 (23.5)	<0.001	51.5 (14.5)	60.9 (13.5)	69.1 (11.2)	85.5 (23.7)	<0.001
Fat (g/day)	73.5 (26.7)	84.7 (32.1)	97.3 (36.4)	104.6 (43.6)	<0.001	63.1 (23.3)	70.6 (26.9)	74.9 (28.1)	88.6 (42.5)	<0.001
Energy consumption (kcal)	1968 (408)	2389 (437)	2700 (469)	3195 (599)	<0.001	1639 (367)	1976 (342)	2211 (344)	2707 (596)	<0.001
Age (years)	51.6 (14.1)	49.6 (13.7)	48.4 (13.0)	48.8 (13.0)	0.13	50.2 (14.8)	48.3 (13.9)	47.0 (12.1)	46.9 (12.2)	0.042
BMI (kg/m^2^)	23.8 (2.9)	23.6 (3.6)	23.1 (3.0)	22.7 (3.1)	0.013	23.5 (3.6)	23.3 (3.9)	23.9 (3.6)	23.7 (3.3)	0.29
Income (%)					<0.001					<0.001
Low	20.4	15.5	18.9	37.9		14.9	21.7	20.3	46.6	
Medium	32.7	39.8	32.1	27.3		38.0	38.2	38.6	24.8	
High	46.9	44.7	49.1	34.8		47.1	40.1	41.1	28.6	
Education (%)					0.19					0.004
Primary school	44.2	35.6	33.7	45.1		55.0	52.6	61.7	71.6	
Junior middle school	38.7	47.2	45.4	40.1		31.1	36.4	27.8	23.1	
Senior middle school	11.7	14.7	17.8	13.0		12.9	10.0	10.5	4.8	
University	5.5	2.5	3.1	1.9		1.0	1.0	0.0	0.5	
Smoking (%)	58.9	61.3	55.8	64.8	0.40	2.9	3.8	1.0	4.3	0.19
Alcohol consumption (%)					0.002					0.17
Never	62.0	50.3	43.8	40.4		95.7	97.1	94.7	91.9	
1–2/week	11.7	14.1	8.0	12.4		1.4	1.9	1.4	1.9	
3–4/week	7.4	7.4	8.6	8.7		1.0	1.0	1.9	1.4	
Daily	19.0	28.2	39.5	38.5		1.9	0.0	1.9	4.8	
Sedentary activity (%)					0.057					0.23
<1 h	11.0	6.7	8.6	16.7		14.4	19.6	19.1	23.0	
1–2 h	23.9	30.1	24.5	32.1		27.8	30.1	33.5	34.0	
2–3 h/day	48.5	47.9	47.2	39.5		43.1	39.7	36.8	33.5	
≥3 h/day	16.6	15.3	19.6	11.7		14.8	10.5	10.5	9.6	
Manual occupation (%)	34.0	50.3	52.8	63.0	<0.001	28.2	50.7	54.1	66.0	<0.001
Region (%)					<0.001					<0.001
Urban	36.2	18.4	12.3	9.3		34.4	17.2	9.1	8.1	
Rural	63.8	81.6	87.7	90.7		65.6	82.8	90.9	91.9	

^1^ Participants presented were those with magnesium measurements at baseline (*n* = 2002) and participated in the follow-up (2007). Data are presented as mean (SD) for such values. * brackets after mean (SD) present magnesium consumption range in each quartile; BMI, body mass index, SD, standard deviation.

**Table 2:** Sex-specific sleep outcomes of subjects by quartiles of magnesium consumption at baseline (2002) (*n* = 1487) ^1^.

Quartiles of Magnesium Consumption (mg/day)										
	Men					Women				
Sleep variables	Q1 (*n* = 163)	Q2 (*n* = 163)	Q3 (*n* = 163)	Q4 (*n* = 162)	*p*-Value	Q1 (*n* = 209)	Q2 (*n* = 209)	Q3 (*n* = 209)	Q4 (*n* = 209)	*p*-Value
Sleep duration (%), (baseline)										
7–8 h/day	64.2	77.0	68.3	64.2	0.19	71.2	69.9	66.7	67.0	0.11
<7 h/day	16.7	11.2	13.0	16.0		13.5	13.1	9.7	8.6	
≥9 h/day	19.1	11.8	18.6	19.8		15.4	17.0	23.7	24.4	
Sleep duration (%), (follow-up)					0.27					0.039
7–8 h/day	74.2	65.4	70.6	73.5		65.1	63.5	65.6	72.2	
<7 h/day	17.8	21.0	16.0	19.1		25.4	20.7	18.7	13.4	
9–13 h/day	8.0	13.6	13.5	7.4		9.6	15.9	15.8	14.4	
Falling sleep (%)	4.9	8.6	5.0	3.7	0.24	8.7	4.4	5.3	1.9	0.018
Daytime sleepiness (%)	12.3	14.7	14.3	13.6	0.92	15.9	13.0	9.6	12.9	0.29
Snore (%)	47.5	51.5	49.7	46.3	0.79	26.8	26.6	26.3	22.5	0.71

^1^ Participants presented were those with magnesium measurements at baseline (*n* = 2002) and participated in the follow-up (2007). Data are presented as mean (SD) for such values.

**Table 3:** The odds ratios (OR (95% CI)) of self-reported sleep disorder symptoms at follow-up (2007) according to magnesium consumption (mg) quartiles at baseline (2002) (*n* = 1487) ^1^.

		Magnesium Consumption (mg/day)			
Sleep disorder symptoms		Men			
Falling asleep	*n*	Q1 (*n* = 163)	Q2 (*n* = 163)	Q3 (*n* = 163)	Q4 (*n* = 162)
Model 1	645	1.00	1.89 (0.73, 4.86)	1.07 (0.35, 3.34)	0.80 (0.20, 3.22)
Model 2	635	1.00	2.32 (0.86, 6.27)	1.32 (0.40, 4.34)	1.38 (0.33, 5.73)
Model 3	607	1.00	2.53 (0.93, 6.90)	1.32 (0.40, 4.34)	1.44 (0.34, 6.04)
Model 4	606	1.00	2.51 (0.91, 6.91)	1.20 (0.36, 4.06)	1.36 (0.31, 5.98)
		Women			
		Q1 (*n* = 209)	Q2 (*n* = 209)	Q3 (*n* = 209)	Q4 (*n* = 209)
Model 1	831	1.00	0.40 (0.17, 0.94) *	0.43 (0.18, 1.05)	0.10 (0.02, 0.43) **
Model 2	795	1.00	0.35 (0.14, 0.87) *	0.41 (0.16, 1.04)	0.10 (0.02, 0.47) **
Model 3	795	1.00	0.32 (0.13, 0.81) *	0.37 (0.14, 0.97) *	0.09 (0.02, 0.43) **
Model 4	792	1.00	0.34 (0.14, 0.87) *	0.42 (0.16, 1.09)	0.11 (0.02, 0.57) **
		Men			
Daytime sleepiness	*n*	Q1 (*n* = 163)	Q2 (*n* = 163)	Q3 (*n* = 163)	Q4 (*n* = 162)
Model 1	649	1.00	1.30 (0.70, 2.42)	1.34 (0.68, 2.63)	1.36 (0.61, 3.03)
Model 2	639	1.00	1.33 (0.71, 2.49)	1.38 (0.69, 2.78)	1.54 (0.68, 3.49)
Model 3	638	1.00	1.33 (0.70, 2.50)	1.41 (0.70, 2.83)	1.64 (0.72, 3.70)
Model 4	637	1.00	1.32 (0.69, 2.50)	1.39 (0.68, 2.83)	1.62 (0.69, 3.80)
		Women			
		Q1 (*n* = 209)	Q2 (*n* = 209)	Q3 (*n* = 209)	Q4 (*n* = 209)
Model 1	834	1.00	0.84 (0.50, 1.43)	0.64 (0.34, 1.17)	0.87 (0.43, 1.73)
Model 2	825	1.00	0.83 (0.49, 1.42)	0.63 (0.34, 1.17)	0.98 (0.47, 2.02)
Model 3	825	1.00	0.83 (0.48, 1.42)	0.62 (0.33, 1.16)	0.99 (0.48, 2.05)
Model 4	821	1.00	0.84 (0.49, 1.44)	0.61 (0.33, 1.14)	1.00 (0.47, 2.08)
		Men			
Snoring	*n*	Q1 (*n* = 163)	Q2 (*n* = 163)	Q3 (*n* = 163)	Q4 (*n* = 162)
Model 1	645	1.00	1.12 (0.81, 1.55)	1.11 (0.78, 1.58)	1.06 (0.69, 1.61)
Model 2	635	1.00	1.06 (0.76, 1.47)	1.07 (0.74, 1.55)	1.11 (0.72, 1.71)
Model 3	607	1.00	1.05 (0.75, 1.47)	1.10 (0.76, 1.59)	1.15 (0.74, 1.80)
Model 4	607	1.00	1.04 (0.75, 1.46)	1.08 (0.74, 1.56)	1.10 (0.70, 1.75)
		Women			
		Q1 (*n* = 209)	Q2 (*n* = 209)	Q3 (*n* = 209)	Q4 (*n* = 209)
Model 1	834	1.00	0.95 (0.65, 1.40)	0.93 (0.61, 1.40)	0.71 (0.42, 1.20)
Model 2	825	1.00	0.97 (0.65, 1.43)	0.94 (0.62, 1.44)	0.80 (0.46, 1.37)
Model 3	825	1.00	1.00 (0.68, 1.49)	0.91 (0.59, 1.39)	0.85 (0.50, 1.46)
Model 4	821	1.00	1.01 (0.68, 1.49)	0.91 (0.60, 1.39)	0.89 (0.52, 1.55)

^1^ Participants included in the model had magnesium consumption at baseline (2002) and participated at follow-up (2007) (*n* = 1487, men (*n* = 651)). Q1–Q4 stands for quartiles of isoflavone consumption for each sex. Model 1 adjusted for age and energy consumption; Model 2 further adjusted for income, education, rural region, smoking, alcohol consumption and sedentary activity; Model 3 further adjusted for BMI, hypertension, and diabetes. Model 4 further adjusted for dietary patterns; Four dietary patterns (previously published) are: Factor 1 (‘macho’) included various kinds of animal foods and alcohol; Factor 2 (the ‘traditional’ pattern) loaded heavily on rice, fresh vegetables and inversely on wheat flour; Factor 3 (‘sweet tooth’) contained cake, milk, yoghurt and drinks; and, Factor 4 (‘vegetable rich’ pattern) was characterized whole grains, fruits, root vegetables, fresh and pickled vegetables, milk, eggs and fish. The four factors explained 28.5% of the variance in intake (published earlier [16]). * *p* < 0.05, ** *p* < 0.01.
